# Facilitating Large‐Scale Bird Biodiversity Data Collection in Citizen Science: ‘Relaxed’ Point Counts for Anytime, Anywhere Monitoring

**DOI:** 10.1002/ece3.72176

**Published:** 2025-09-25

**Authors:** Masumi Hisano

**Affiliations:** ^1^ Graduate School of Informatics Kyoto University Kyoto Japan; ^2^ Global Change and Biodiversity Lab, Graduate School of Advanced Science and Engineering Hiroshima University Higashihiroshima Japan

**Keywords:** avian species assemblage, big data collection, biodiversity monitoring, field bird survey, public participation in science, spatial autocorrelation, species distribution

## Abstract

Citizen science has expanded biodiversity monitoring, yet many datasets lack standardisation in spatial and temporal coverage and survey protocols. In birds, for example, traditional point count surveys often impose strict requirements on location, timing and spacing between survey points, limiting opportunities for casual, at‐ease participation in data collection. To address these constraints, this paper proposes a ‘relaxed’ point‐count survey method to enhance accessibility and expand geographic coverage by easing these constraints. Surveys can be conducted in diverse locations, including urban areas and travel or daily‐routine routes, within flexible timeframes (e.g., not only within 6 h after sunrise but also afternoon/evening) and seasons (e.g., including non‐breeding periods), with adaptable spacing between points and the option for repeated counts at the same location on different days. The framework addresses spatial and temporal autocorrelation, as well as variability in observer skill and environmental conditions through statistical adjustments using random effects and covariates. Preliminary data collected opportunistically across a large area of western Canada demonstrate the feasibility of this approach, yielding cross‐biome community data within a short timeframe. By engaging birdwatchers and citizens, this approach facilitates the collection of large‐scale, standardised species assemblage data beyond single‐species observations. This inclusive and scalable strategy offers new opportunities for biodiversity monitoring, particularly in human‐modified landscapes. This inclusive and scalable framework offers new opportunities for biodiversity monitoring, particularly in urban and human‐modified landscapes.

## Graphical abstract

This paper proposes a ‘relaxed’ point‐count survey method to enhance accessibility and expand geographic coverage by easing these constraints. Surveys can be conducted in diverse locations, including urban areas and travel routes, within flexible timeframes (e.g., 6 h after sunrise or during the evening) and with adaptable spacing between survey points, with repeated counts at the same location on different days. The ‘relaxed’ point count survey offers a scalable, inclusive framework for avian biodiversity monitoring, particularly in urban and understudied landscapes, contributing to further understanding of bird community dynamics.

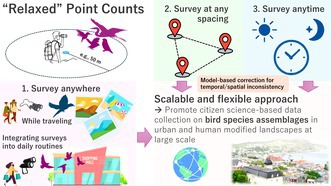

## 市民科学开展大规模鸟类生物多样性数据收集：灵活化定点计数调查，实现随时随地监测

市民科学在促进生物多样性监测方面取得了显著进展并不断拓展，但许多数据集在空间与时间覆盖以及调查程序标准化方面仍然不足。以鸟类为例，传统的定点调查法往往对调查地点的位置、时间和间隔设定严格限制，从而减少了公众轻松参与市民监测的机会。为缓解这些限制，本文提出一种“宽松型”定点普查法，通过放宽调查要求，提高参与便利性，并在地理上扩大鸟类群落数据的采集范围。该方法可在多样化的地点开展调查，包括城市区域和移动路线(如旅行、日常生活、通勤途中等)，可在灵活的时间段(不仅限于日出后6小时内，还包括下午或傍晚)和季节(包括非繁殖期)实施，调查点间的间距亦可自由调整，同一地点亦可在不同日期重复开展调查。针对空间与时间自相关、调查者技能差异以及气候与环境条件等问题，本方法建议通过引入随机效应和时间协变量的统计模型加以处理。此外，本文还介绍了在加拿大西部大范围地区以机会性方式(顺带其他目的)收集的初步数据，以展示该方法的可行性，并表明在短短数天内即可获得跨越多个生物群系的鸟类群落数据。通过吸引观鸟者和公众参与，该方法能够收集大规模、标准化的生物群落数据。这一灵活且可扩展的框架为生物多样性监测带来了新的机遇，尤其适用于城市及高度人类改造景观的广域应用。

## 市民科学における大規模鳥類生物多様性データ収集の促進：いつでもどこでもできる"ゆる"スポットセンサスのすすめ

市民科学は生物多様性モニタリングを大きく進展・拡大させてきたものの、多くのデータセットは依然として、空間的および時間的な包括性や調査手順の標準化が不十分である。例えば鳥類では、従来の定点調査法(以下、スポットセンサス法)が調査地点の位置・時間・間隔に厳しい制約を課すことが多く、気軽に参加できる市民モニタリングの機会を制限してきた。こうした制約を和らげるため、本稿では「緩やかな」スポットセンサス法(“ゆる調査”)を提案し、調査要件を緩めることで参加のしやすさを高め、鳥類群集データの収集範囲を地理的に広げることを目指す。本方法では、都市部や移動経路上(旅行中や日常生活、通勤途中など)の多様な場所で調査を行うことができ、柔軟な時間帯(例:日の出後6時間以内のみに限らず、午後や夕方も)や季節(例:非繁殖期も含める)で実施可能であり、調査地点間の間隔も任意に調整可能とし、同一地点でも日を変えて繰り返し調査することも可能とする。空間的・時間的自己相関や、調査者技能の差、および気候・環境条件の違いに対応するランダム効果や時間的な共変量を用いた統計手法についても触れる。さらに、カナダ西部の広域で機会的に(他の目的のついでに)収集した予備データを紹介することで、本手法の実現可能性を示し、数日という短期間で複数のバイオームにまたがる鳥類群集データを得られることを実証した。バードウォッチャーや市民の参加を得ることで、大規模かつ標準化された生物群集データの収集が可能になる。柔軟で拡張性高い本枠組みは、生物多様性モニタリングに新たな可能性をもたらし、とくに都市や人為改変の進んだ景観における広域的な活用が期待される。

## 1 Introduction

In recent years, the development of citizen science, i.e., research conducted with the voluntary participation of non‐professionals in scientific data collection, has facilitated the accumulation of biodiversity data (Bonney et al. 2009; Dickinson et al. 2010; Fraisl et al. 2022). Citizen science‐driven data can be largely classified into unstructured (e.g., casual observations), semi‐structured (e.g., checklists with optional effort information) and structured forms (e.g., standardised protocols with fixed ranges and times) (Kelling et al. 2019), which influence data quality and suitability depending on objectives and analyses taken. Compared to structured monitoring led by experienced researchers, many citizen science initiatives, especially those in the unstructured or semi‐structured categories, often allow more flexible data collection in terms of timing, location and observer training, aiming to engage a broader audience (Bonney et al. 2009; Kelling et al. 2019). Birds, in particular, attract great public interest due to their appealing nature and the relatively less identification skills required, which has resulted in an extensive repository of information. Platforms such as eBird (Sullivan et al. 2009, 2014) allow users to upload and share data on bird sightings and photographs, making it easy to provide and access observational records. Building on such foundational tools, more recent smartphone applications (e.g., Catlin‐Groves 2012; Lee and Nel 2020) have introduced enhanced, user‐friendly mapping interfaces and real‐time spatial feedback, making it easier for users to plan and adjust surveys on‐site. These newer tools often support intuitive map‐based inputs and offline functionality. Together, these developments have expanded both the accessibility and spatial accuracy of citizen‐collected biodiversity data (Jäckel et al. 2021).

Many citizen science datasets, however, remain limited in standardisation, particularly in spatiotemporal coverage and survey protocols. While some platforms like eBird allow observers to record exact counts in addition to simple presence/absence, most of them do not follow fixed‐duration or fixed‐radius survey protocols. As a result, the data are often semi‐structured and vary in effort, area covered and detectability, making it difficult to interpret them as standardised species assemblage datasets suitable for community‐level comparisons. In contrast, many governmental and volunteer‐based national or regional citizen science initiatives do conduct standardised bird community surveys using fixed‐point counts or transects [e.g., North American Breeding Bird Survey (Ziolkowski et al. 2023), Wild bird populations in the UK and in England (Department for Environment 2024), the French Breeding Bird Survey (Jiguet et al. 2012), Swiss Breeding Bird Atlas (Knaus et al. 2018), Japanese Monitoring Sites 1000 Satoyama Birds (Biodiversity Center of Japan 2014), the Taiwan Breeding Bird Survey (Ko et al. 2014)]. Nevertheless, these efforts are often limited to activities conducted by nature conservation groups registered with government monitoring programs. As a result, broader public participation in collecting standardised bird community data remains restricted.

**FIGURE 1:**
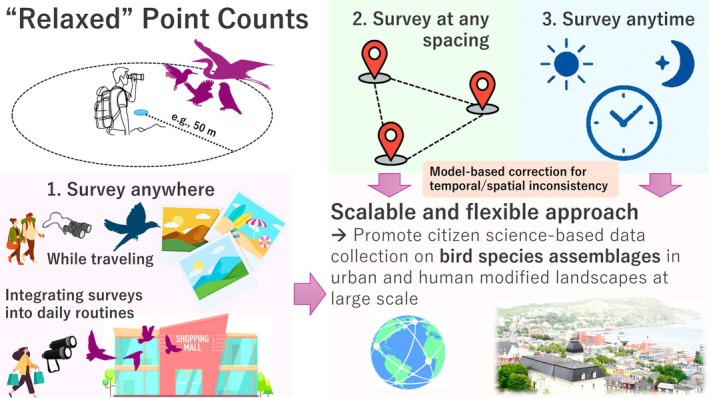
Schematic summary of the relaxed point‐count method, comprising three components: (1) surveys conducted anywhere and integrated into daily routines; (2) flexible spacing between points; and (3) surveys at any time. Methodological inconsistencies should be addressed using model‐based statistical approaches. The figure materials are based on free images available from *Canva* (https://www.canva.com) and the photograph is credited to Masumi Hisano.

To overcome these limitations, it is necessary to develop a bird community data collection scheme that is accessible, convenient and can be conducted anytime and anywhere without psychological or physical barriers. In response, I propose a ‘relaxed’ point count survey as a more flexible citizen‐participatory bird monitoring approach (Figure 1). This method is not about developing a new survey technique but rather about easing the stringent conditions traditionally associated with point count surveys set by researchers. By reducing the psychological and physical burden of these surveys, this approach aims to make participation more feasible for a broader audience. This paper explores the potential of utilising observations during our daily lives by relaxing constraints on location, time and frequency, thereby enabling more extensive and long‐term bird monitoring. While ensuring data quality, it acknowledges that variability may occur under certain conditions. The aim is to shift perspectives on conducting surveys, encouraging a more accessible and participatory approach and expanding the sample size and geographic coverage. Lastly, I present an example dataset collected using the proposed ‘relaxed’ point count method and demonstrate how surveys can be seamlessly integrated into personal daily routines, travel, or other activities, illustrating the practical application and potential scalability of this approach.

## 2 Relaxation of Spatial Constraints: Surveying Anywhere

Ecologists often conduct surveys in remote mountains or agricultural lands, requiring researchers to travel far from their institutes or homes. However, valuable data may also be embedded in our daily environments. So far, extensive bird community monitoring data has been collected in forests, farmlands and shore and wetland habitats (e.g., Jiguet et al. 2012; Biodiversity Center of Japan 2014; Ko et al. 2014; Knaus et al. 2018; Ziolkowski et al. 2023; Department for Environment 2024). However, while population‐level monitoring data is relatively abundant in urban ecosystems, community‐level data remains comparatively scarce (Veech et al. 2012; Fidino and Magle 2017). This perspective also extends to other human‐modified environments such as nature‐based tourism sites, where systematic data on avian communities are still limited despite increasing human influence. To fill in this data gap, point counts can be conducted in residential areas, along commuting routes, in workplace greenspaces, or even in supermarket parking lots (Figure 1). Similarly, surveys can be performed in urban areas during business trips or private visits to tourism destinations, which can contribute valuable observations. Urban greenspaces and water bodies, which often provide various bird species, are widespread. Collecting data in fully urbanised landscapes without habitat patches is also crucial for capturing matrix conditions, particularly for studies on habitat fragmentation and ‘land sparing vs sharing’ (Soga et al. 2014; Ibáñez‐Álamo et al. 2020). Therefore, data from completely urban landscapes can be essential controls in such research.

**FIGURE 2:**
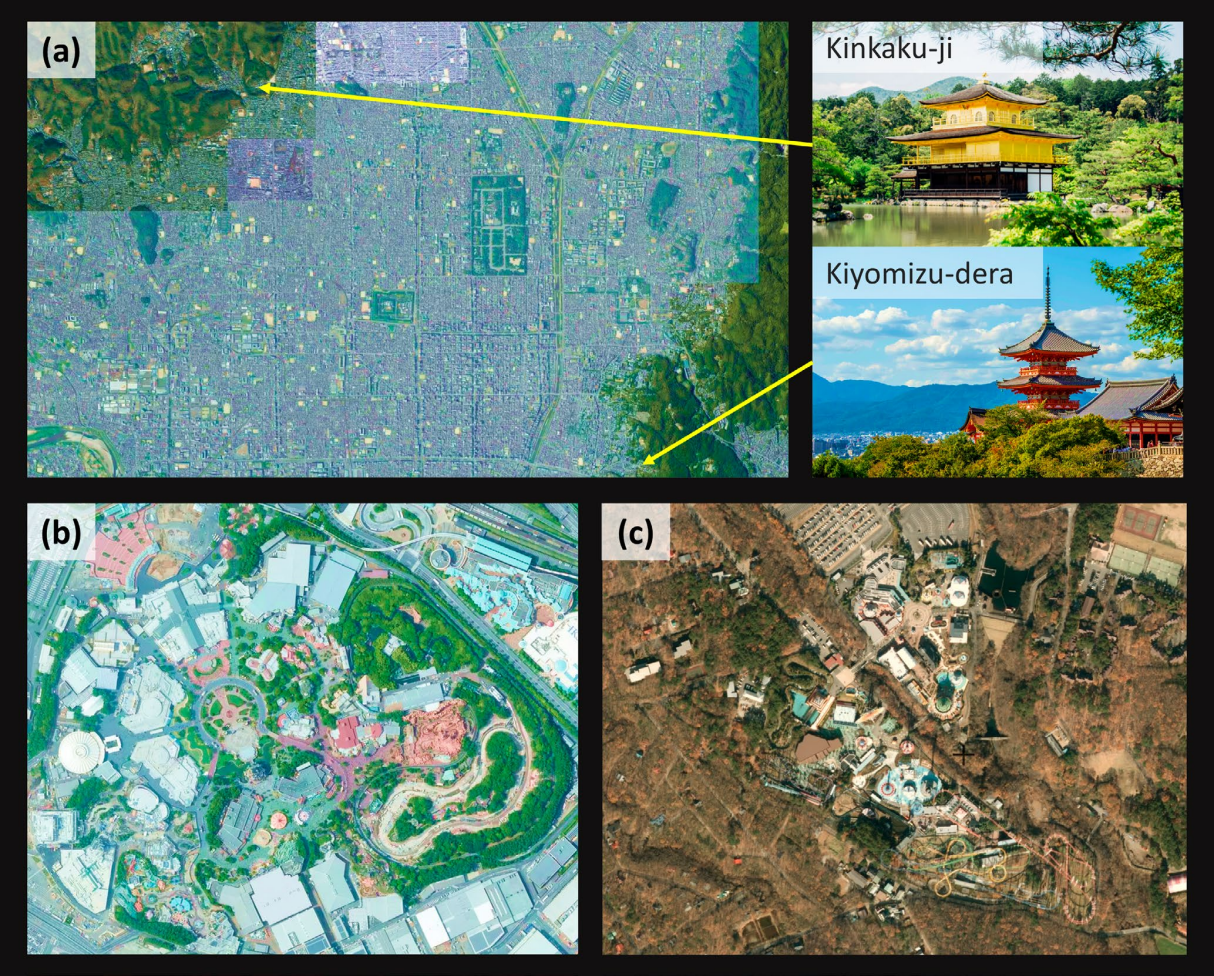
Viewing tourism sites through a landscape ecology perspective. (a) Urban landscape of Kyoto (western Japan), one of the world's major tourist destinations, characterised by small patchy urban greenspaces and sacred shrine forests that function as important habitats for bird assemblages (Hamada and Fukui 2013; Skórka et al. 2018; Matsumoto et al. 2024). Locations of two example landmarks (Kiyomizu‐dera and Kinkaku‐ji) are shown with yellow arrows, illustrating the surrounding habitat and suggesting how popular tourist sites may also provide suitable environments for birds. (b) A popular urban theme park in the Tokyo Metropolitan, central Japan, incorporating planted urban greenspaces. (c) A regional theme park in Tochigi Prefecture, central Japan, developed through the fragmentation of natural forests. The figure materials are based on the aerial photos of Geospatial Information Authority of Japan (https://www.gsi.go.jp/; photographed in 2020) and free images available from *Canva* (https://www.canva.com).

More importantly, this mindset is beneficial not only in daily life but also during travel and tourism. For example, point counts can be conducted at highway rest stops while travelling. In the world's popular tourist destinations, street trees (Wood and Esaian 2020) or sacred forests (Skórka et al. 2018; Matsumoto et al. 2024) may provide suitable sites for bird community surveys. For example, studies could be conducted in the old town areas and shrine forests of Kyoto (Hamada and Fukui 2013) as part of tourism activities (Figure 2a). Once travelling outside urban areas, farmland, secondary forests, as well as their mosaic landscapes often become accessible, offering opportunities for bird community data collection in these environments. In mountainous tourist destinations, hiking can provide data from more intact natural settings. Surveys can also be carried out in theme parks, which often incorporate trees and greenspaces (Figure 2b), making urban greenspace sampling effectively feasible. Many regional theme parks are developed by clearing original forests or mountainous areas (Figure 2c). Bird community data from these landscapes are invaluable for landscape ecological studies, particularly those examining the impacts of habitat fragmentation. The key perspective here is to integrate fieldwork into (even personal) travel rather than adhering to the conventional approach of ecologists travelling solely for research. By shifting the mindset to conducting fieldwork as part of private leisure and tourism activities, researchers can enhance data collection while increasing public engagement in ecological studies.

## 3 Relaxation of Distance Constraints: Surveying at Any Spacing, While Minimising Autocorrelation

In bird point count surveys, maintaining a certain distance between survey points is commonly recommended to avoid both pseudoreplication and spatial autocorrelation, which are related but distinct concepts. Pseudoreplication occurs when samples from the same population are not statistically independent, which can occur due to temporal or spatial proximity of sampling units. Spatial autocorrelation refers to the phenomenon where nearby survey points are more likely to yield similar observations due to shared environmental or habitat characteristics, which may contribute to pseudoreplication in statistical analyses. To mitigate this issue, traditional protocols often apply a minimum distance threshold (typically 300 m to 1 km) between survey points (e.g., Lituma and Buehler 2016; Boesing et al. 2018; Ziolkowski et al. 2023). Alternatively, points may be randomly or strategically subsampled to reduce spatial clustering. However, in opportunistic or semi‐structured surveys, such as those conducted near one's home, workplace, or along commuting routes, maintaining such spatial constraints can be impractical, potentially limiting sample size and coverage.

In addition to spatial concerns, pseudoreplication may also arise from temporal autocorrelation, especially when the same location is surveyed repeatedly over short intervals. In stable bird communities, particularly during breeding or wintering seasons, observations on consecutive or closely spaced days may lead to re‐detection of the same individuals. This temporal dependence can bias abundance or occupancy estimates if independence is assumed. Although some studies (e.g., Drapeau et al. 1999; Hisano et al. 2025a) have incorporated repeated visits as a way to assess detection reliability, such designs typically treat multiple surveys as temporal replicates rather than independent samples.

**FIGURE 3:**
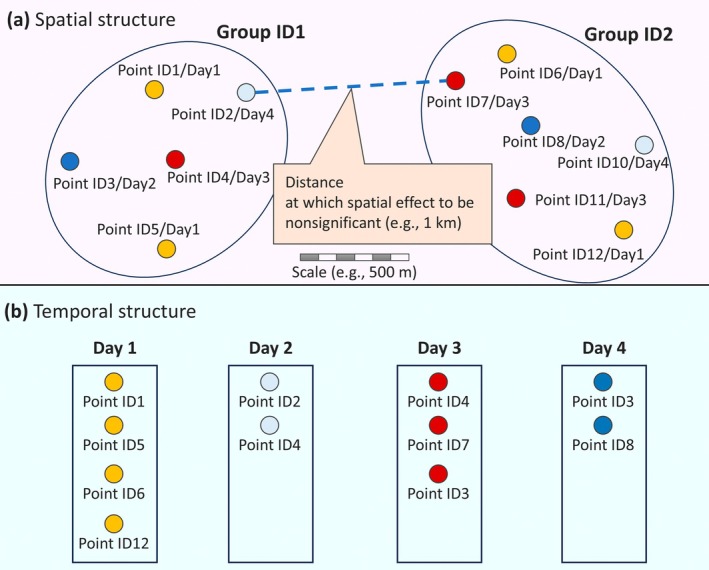
Schematic illustration of spatial and temporal grouping for addressing autocorrelation in bird point count surveys. (a) Spatial structure: Survey points located within a threshold distance at which spatial autocorrelation becomes negligible [e.g., 1 km here as an example, which should be determined using statistics such as Moran's I (e.g., Aguirre‐Gutiérrez et al. 2022; Ding et al. 2024; Hisano et al. 2024)] are assigned a shared Group ID. These groups may include points surveyed on different days. To avoid pseudoreplication, surveys should also follow minimum separation rules [e.g., ≥ 500 m as an example here, depending on study assumptions (e.g., Lituma and Buehler 2016; Boesing et al. 2018; Ziolkowski et al. 2023)]. In this example, points surveyed on the same day include Point IDs 1, 5, 6 and 12, each separated from the others by at least 500 m. (b) Temporal structure: Survey days (Day 1–Day 4) represent non‐consecutive dates separated by certain intervals (e.g., 7 days, subject to validation). Points surveyed on the same date share the same Day label. By combining both Group ID (spatial) and Day (temporal) as random effects, it is suggested to account for non‐independence arising from proximity in space and time, thereby maximising sample size while easing spatial and temporal restrictions.

Thus, treating each day as a fully independent observational unit should be done cautiously. The appropriate temporal interval between surveys—whether 3, 7, or more days—should ideally be determined based on empirical evidence of community turnover or species mobility. In the absence of such data, a more conservative approach is to model temporal autocorrelation explicitly, for instance by including ‘Date’ (or visit ID) as a random effect (Figure 3b) to account for date‐specific influences such as weather (Ellis and Taylor 2018), or by using time‐series structures [e.g., autoregressive models (Ver Hoef et al. 2018)]. Similarly, when full spatial independence between points cannot be achieved, statistical adjustments can help mitigate spatial autocorrelation. For repeated visits to the same site, ‘Point ID’ can be modelled as a random effect (Koshkin et al. 2016; Guélat and Kéry 2018; Hisano et al. 2025a) to control for unmeasurable microsite‐specific conditions. If nearby points are visited on different days and fall within a distance where spatial autocorrelation is likely, they may be assigned a shared ‘Group ID’ and included in the model as a random effect (Figure 3a) to account for regional factors such as local geoclimatic conditions or habitat type. This process involves (i) testing model residuals for spatial dependence (e.g., using Moran's I), (ii) estimating the distance at which this dependence becomes negligible and (iii) grouping points within that distance under a common ‘Group ID’ (Figure 3). A similar procedure has been used in forest inventory analyses (Aguirre‐Gutiérrez et al. 2022; Ding et al. 2024); however, to my knowledge, its application to bird point‐count surveys remains underexplored. Besides that, determining an appropriate interval (e.g., whether 3, 7, or more days) should ideally be grounded in empirical evidence (Figure 3b), presenting a direction for future research.

In summary, by addressing pseudoreplication and spatial/temporal autocorrelation through thoughtful temporal separation and statistical adjustments, the emphasis should be on maximising sample size through flexible survey practices rather than strictly adhering to distance constraints. In addition to the schemes proposed above, several tools and frameworks can help address such methodological concerns, including the R packages of *blockCV*, which facilitate spatial cross‐validation in species distribution models (Valavi et al. 2019, 2023), and *DynamicSDM*, a framework designed to correct spatial and temporal biases in unbalanced datasets (Dobson et al. 2023).

## 4 Relaxation of Time Constraints: Surveying Anytime

Bird activity is generally highest during the first few hours after dawn, especially during the breeding season (Järvinen et al. 1977; Esquivel and Peris 2008) when vocalisations increase detection rates. Consequently, many studies conducted surveys within 3–4 h after sunrise (Boesing et al. 2018; Deguchi et al. 2020; Morante‐Filho et al. 2021). However, early morning surveys can be burdensome due to personal health or lifestyle rhythms. A recent study in temperate forests of Argentina demonstrated that bird species richness and abundance do not significantly differ within 6 h post‐sunrise compared to earlier periods (Frutos et al. 2019). In contrast, other regions such as temperate *Eucalyptus* forests in southeastern Australia (Slater 1994; Ellis and Taylor 2018) and subtropical moist forests in Paraguay (Esquivel and Peris 2008) show higher detection rates shortly after sunrise, while temperate oak–pine woodlands in western North America show no strong temporal pattern (Verner and Ritter 1986). Other research has found trends dependent on species across North America (Robbins 1981) or on habitat type in the Czech Republic (Morelli et al. 2022). Although broader validation across biomes is needed, these studies suggest that extending survey windows up to 6 h post‐sunrise may not substantially compromise data quality in certain climates or habitats (Verner and Ritter 1986; Frutos et al. 2019; Morelli et al. 2022); also supported by analyses of open data from Hisano et al. (2025a, b) for farmlands in central Japan. If this pattern holds in a given study region, relaxing time‐of‐day restrictions could ease participation and better align with monitoring participants' daily routines. Most importantly, surveying across the full morning period, including peak activity times, allows for statistical control of time effects by incorporating ‘time since sunrise’ or time‐interval covariates into models. Indeed, several studies have successfully accounted for within‐day temporal variation by including ‘time since sunrise’ as a covariate (e.g., Lituma and Buehler 2016; Edwards et al. 2023).

Moreover, I advocate for the acceptance of evening surveys when appropriate. A study conducted in a tropical urban greenspace in Indonesia using point count surveys showed negligible differences in avian species diversity and abundance between morning (6:00–8:00 a.m.) and evening (3:00–5:00 p.m.) (Nugroho et al. 2021). However, further verification is needed for generalisation, as other studies have found significantly higher bird abundance detection in the early morning (4:00–8:00 a.m.) compared to other time periods, including evening, in boreal forests and fields in Finland (Järvinen et al. 1977). Nevertheless, even if data completeness and standardisation might be compromised, modelling detection rates across different time periods to allow for robust statistical corrections (mentioned above) remains valuable. This is particularly important for detecting crepuscular species. Since most breeding bird surveys are conducted in the morning, expanding survey times could help capture a broader range of species; for example, in eastern Brazil, nearly half of the raptor species were detected only during afternoon hours (Oliveira et al. 2018). Therefore, I propose a less constrained approach regarding the time of day, encouraging flexibility in timing to maximise bird observation sampling across a broader temporal scale within a day. This would eliminate the restriction of conducting surveys exclusively in the early morning, making it easier to align survey times with our daily activities. Consequently, this approach would facilitate the integration of point count surveys into our everyday routines.

Regarding survey duration, point counts are conducted for 15–30 min in many studies (e.g., Peh et al. 2005; Croci et al. 2008; Sirami et al. 2009; Boesing et al. 2018; Morante‐Filho et al. 2021). However, the majority of bird observations occurred within the first 5–10 min of the survey in temperate forests of Paraguay (Esquivel and Peris 2008) and France (Bonthoux and Balent 2012). On the basis of this, reducing the survey duration to 5 or 10 min (Monroe et al. 2021) can be considered [even 3 min are employed in the North American Breeding Bird Survey (Ziolkowski et al. 2023), although with a 400 m radius for a survey range]. Shorter surveys not only minimise the risk of double counting within a single session (Matsuoka et al. 2012) but also make data collection easier to integrate into daily routines while reducing the burden on surveyors. The adequacy of shorter durations (e.g., 3, 5, or 10 min) versus longer ones (> 15 min) in effectively capturing regional avifauna remains unclear and requires empirical evaluation across different regions and biomes. Nonetheless, the critical point is maintaining consistency in survey duration across all sites to ensure data reliability (Shen et al. 2023). By relaxing time constraints, survey flexibility can increase, allowing for the collection of a greater number of samples.

Additionally, relaxation of seasonal constraints should also be considered. Standardised bird monitoring programs, such as the North American Breeding Bird Survey (U.S. Department of the Interior 2023) or national censuses in many countries or regions (e.g., Ko et al. 2014; Heywood et al. 2025), typically focus on the breeding season due to its biological relevance, when birds are most vocal, territorial and site‐faithful, making detection more consistent and population estimation more reliable. However, this seasonal restriction limits our understanding of avian ecology to a narrow temporal window, often overlooking patterns and processes occurring outside the breeding season, such as wintering and migratory seasons (Latta and Faaborg 2009; Dybala et al. 2015).

## 5 Preliminary Data From the ‘Relaxed’ Anytime–Anywhere Approach

Applying the proposed ‘relaxed’ approach, large‐scale, cross‐biome data on avian species assemblages were obtained opportunistically during a single week in summer in Alberta, western Canada (Hisano 2025). While coupled with research expeditions and fieldwork conducted for other purposes, point counts were conducted at 56 sites without prior site selection, reflecting the ‘anytime–anywhere’ principle. Survey locations encompassed a range of elevations (678–1623 m a.s.l.) and spanned Alberta's six major ecoregions (Olson et al. 2001), representing three biomes (temperate, prairie and boreal), across multiple habitat types including urban areas, forests and farmlands. Sites also included unconventional settings, including parking areas at shopping centres, highway rest stops, sightseeing locations, accommodation grounds and high‐profile destinations (e.g., Calgary, Banff National Park). Surveys were conducted at various times of day from morning to sunset, and time‐of‐day data were recorded (Hisano 2025) to enable subsequent modelling of detection probability relative to time since sunrise (Lituma and Buehler 2016; Edwards et al. 2023). In total, 324 individuals of 48 avian species were recorded. This dataset illustrates how spatial and temporal flexibility can maximise data yield, covering a large‐scale area [approximately 8.4 million ha; Figure 1 in (Hisano 2025)] within a short time frame, while reducing logistical and scheduling constraints. This suggests the approach enables casual participation with ease, broadening public engagement in biodiversity monitoring.

## 6 Conclusion and Future Directions

This paper proposes an innovative yet straightforward approach to efficiently collect avian community data through a ‘relaxed’ point count survey method. By easing constraints on location, time and survey point spacing, this approach reduces the burden on surveyors, facilitating the accumulation of large‐scale ecological data. Birdwatchers and naturalists, with their strong identification skills, can play a crucial role in scientific data collection. Many existing citizen science platforms, such as eBird (Sullivan et al. 2009, 2014), already yield valuable community‐level data across regions by encouraging observers to report all species detected and their counts. However, these datasets are often inconsistent in survey duration and radius (or may even lack such constraints), with varying levels of effort, which can introduce variability in detectability and comparability. Encouraging participants to adopt simple, standardised protocols (e.g., fixed survey radius and duration) within such platforms could enhance data consistency, contributing to more robust and geographically extensive datasets of avian species assemblage. In addition, even when conducting ‘relaxed’ point counts, participants and researchers should retain a landscape and macro‐ecological perspective (considering factors such as habitat loss and fragmentation, land‐sparing vs. land‐sharing strategies, connectivity of green spaces, the intensity of human activity, etc.) so that scaled‐up community‐level datasets can meaningfully inform research on biodiversity patterns in urban and other human‐modified landscapes (Dickinson et al. 2010, 2012).

Nonetheless, there are several potential caveats. Participation in citizen science projects tends to be unevenly distributed, with fewer contributors in sparsely populated areas and a tendency to survey easily accessible or perceived species‐rich locations. This spatial bias can lead to uneven data quality and may limit generalisability, particularly in under‐sampled regions (Boakes et al. 2010; De Coster et al. 2015; Planillo et al. 2021). Additionally, variability in observer skills remains also a challenge. However, this can be addressed by incorporating observer expertise (e.g., the numbers of species detected by observers) as a covariate in models (Johnston et al. 2018), or by quantifying behavioural patterns of citizen scientists to identify and correct for systematic biases (August et al. 2020). To ensure the scientific utility of data collected via ‘relaxed’ point counts, future research should focus on empirical validation of these approaches and the development of statistical methods to account for sampling biases, such as effort‐weighted modelling, spatial subsampling and bias‐informed background selection (Bird et al. 2014; Steen et al. 2019).

Despite these challenges, the ‘relaxed’ point count method holds significant potential for expanding avian biodiversity monitoring, particularly in urban and understudied landscapes, if coupled with thoughtful design and analytical rigour. Further empirical verification is required to determine whether this perspective, based on limited evidence, can be effectively applied in real surveys and reliably used in scientific, data‐driven analysis. By integrating surveys into daily life, furthermore, such approaches may foster greater noticing of nearby birdlife among participating citizen scientists, with likely benefits for human well‐being (White et al. 2023; Peterson et al. 2024; Soga et al. 2025).

## Author Contributions

**Masumi Hisano:** conceptualization (lead), data curation (lead), funding acquisition (lead), methodology (lead), project administration (lead), resources (lead), visualization (lead), writing – original draft (lead), writing – review and editing (lead).

## Conflicts of Interest

The author declares no conflicts of interest.

## Data Availability

The dataset associated with this study is published as a data paper in *Ecosismas* (Hisano 2025) and is openly available at *figshare* (https://doi.org/10.6084/m9.figshare.28078583.v1).
